# Can students benefit from prompting during experimentation? An investigation of the benefits of incremental scaffolds compared to sample solutions on cognitive load and flow experience in biology lessons

**DOI:** 10.3389/fpsyg.2026.1754081

**Published:** 2026-04-15

**Authors:** Marlina Hülsmann, Matthias Wilde

**Affiliations:** Faculty of Biology, Biology Didactics, Bielefeld University, Bielefeld, Germany

**Keywords:** incremental scaffolds, sample solutions, prompting, experimentation, cognitive load, flow

## Abstract

**Introduction:**

Previous studies have shown that incremental scaffolds can support learners during the cognitively demanding task of experimentation, both cognitively and affectively. However, it remains unclear which component of these scaffolds is primarily responsible for their effectiveness. To address this gap, the present study compared incremental scaffolds with embedded prompts to sample solutions without prompting to examine the potential added value of prompting within incremental scaffolds.

**Methods:**

This study investigated the effects of incremental scaffolds versus sample solutions on students’ extraneous cognitive load and their flow experience during biology experimentation. A total of 145 high school students (*M*_Age_ = 12.87 years, *SD*_Age_ = 0.64 years) participated in two consecutive experiments on animals’ thermoregulation strategies. The experimental group worked with incremental scaffolds that initially provided a prompt before revealing the sample solution, whereas the control group received sample solutions only.

**Findings:**

In both experiments, participants in the experimental group reported significantly higher flow experiences than the control group. Concerning extraneous cognitive load, the groups did not differ in the first experiment, but in the second, extraneous cognitive load was significantly lower in the experimental group. These findings suggest that the combination of prompts and sample solutions within incremental scaffolds is associated with reducing cognitive load and enhancing students’ perceived learning experience.

## 1 Introduction

As a central method of gaining knowledge in the natural sciences, experimentation plays a crucial role in biology education (Baur and Emden, 2020; Osborne et al., 2003). It enables students to develop both conceptual understanding and inquiry skills such as hypothesizing, planning, and interpreting results (Kremer et al., 2019). Yet experimentation is often cognitively demanding, as learners must coordinate procedural, conceptual, and strategic aspects simultaneously, which can lead to cognitive overload (Kirschner et al., 2006). Such challenges may contribute to difficulties in science learning more broadly, a reality reflected in large-scale assessments such as PISA, which have documented declining performance among German students (Lewalter et al., 2023). These findings highlight a pressing need for instructional approaches that support students during experimentation without oversimplifying the learning process or reducing active engagement. Adequate guidance is therefore essential to reduce excessive cognitive demands while keeping students cognitively and affectively engaged (Belland et al., 2013; Kirschner et al., 2006).

To support the experimentation process, incremental scaffolds provide a promising approach by offering stepwise support during complex tasks (Hänze et al., 2010; Schmidt-Weigand et al., 2008). Typically, they combine prompts, which offer hints or guiding questions to activate prior knowledge and reasoning, with sample solutions that make the correct experimental step explicit. Studies show that incremental scaffolds can foster, e.g., knowledge acquisition (Großmann and Wilde, 2019; Stiller and Wilde, 2021; Hülsmann and Wilde, under review; Mustafa et al., 2021), motivation (Kleinert et al., 2021; Schmidt-Borcherding et al., 2013), and scientific reasoning (Arnold et al., 2017). However, incremental scaffolds have usually been investigated as a whole, leaving open the question of which element accounts for these benefits. As a consequence, it remains unclear to what extent the inclusion of prompts within incremental scaffolds contributes to supporting learning without undermining learners’ active cognitive processing. Prompts are designed to activate learners by encouraging reflection and independent attempts, while sample solutions may reduce uncertainty and ensure correctness (Hänze et al., 2010; Schmidt-Weigand et al., 2008). Nevertheless, the role of prompting within incremental scaffolds and its effects on learners’ cognitive and affective experiences during authentic hands-on experimentation have not yet been systematically examined.

To explore this question, it is necessary to consider not only cognitive performance but also the quality of the learning process. Many prior studies have focused on outcome measures like knowledge acquisition, yet less is known about how students actually experience the experimentation process with incremental scaffolds. Flow—characterized as a state of deep involvement and positive affect (Csikszentmihalyi, 1990, 2010)—has not yet been examined in this context. From an instructional perspective, flow is particularly relevant because it indicates whether learners perceive experimentation tasks as engaging and appropriately challenging rather than frustrating or overwhelming. Flow reflects whether activities are engaging and well-matched to learners’ skills. It provides an important complement to cognitive measures such as extraneous cognitive load (eCL). eCL captures how efficiently cognitive resources are used by indicating the effort spent on irrelevant task designs or demands (Sweller et al., 2019). Flow, in contrast, represents the extent to which learners perceive the activity as optimally involving and challenging (Csikszentmihalyi, 1990, 2010). Examining both constructs together allows for a more nuanced understanding of how instructional scaffolds shape not only learning outcomes but also the quality of the learning experience. Considering both constructs thus offers a more comprehensive view of how incremental scaffolds influence experimentation.

Rather than comparing different teaching approaches, the present study adopts a component-focused perspective within one established instructional framework. Incremental scaffolds were deliberately chosen because they represent a commonly applied and theoretically grounded form of instructional support in science education, yet their internal components are rarely examined separately (Stiller and Wilde, 2021). By disentangling prompts and sample solutions, this study aims to clarify the contribution of these components within incremental scaffolds to leaners’ cognitive and affective experience during experimentation, including extraneous cognitive load and flow experience.

## 2 Theoretical framework

### 2.1 Experimentation

Experimentation plays a key role as a central method of scientific inquiry in science education and should therefore be mastered by students (Baur and Emden, 2020; Osborne et al., 2003; Schwichow et al., 2016). Experimentation provides students with an approach to engage in scientific investigation, helping them understand both the processes and principles underlying scientific knowledge acquisition (Kremer et al., 2019; Lederman, 2009). In this way, students can acquire a comprehensive grasp of the procedures central to scientific thinking and methodology (Wirth et al., 2008). Experimentation has the potential to promote both content knowledge and an appropriate understanding of science and scientific work, as well as the ability to conduct scientific work scientifically independently (Kremer et al., 2019). Furthermore, students often respond positively to experimental activities, which can spark situational interest (Kirchhoff et al., 2023) and may foster a more enduring interest in biology through repeated engagement (Hidi and Renninger, 2006).

During an experiment, the experimenter intentionally manipulates the procedure by controlling relevant conditions, first identifying the dependent variables and then systematically varying the independent variables (Kremer et al., 2019; Schmidt, 2016). Mastering experimentation entails multiple interrelated steps: formulating research questions, generating hypotheses, designing and conducting experiments, collecting and analyzing data, and drawing evidence-based conclusions (Abd-El-Khalick et al., 2004; Kremer et al., 2019). Students often perceive these processes as complex, and deficits in skills such as hypothesis generation, experimental planning and execution and evaluation (Kranz et al., 2022) can lead to cognitive overload (Kirschner et al., 2006). Because experimentation demands both cognitive and practical skills from learners (Hofstein and Lunetta, 2004), instructional support is essential to mitigate cognitive load and help learners navigate the process effectively (Kirschner et al., 2006). One possible approach is incremental scaffolding (Hänze et al., 2010; Leisen, 2003; Schmidt-Weigand et al., 2008).

### 2.2 Instructional support: from worked examples to incremental scaffolds

A variety of instructional support measures can be implemented to facilitate student learning in complex tasks such as experimentation. These strategies can be understood within the broader framework of *scaffolding*, which provides temporary support for learners to help them accomplish tasks that exceed their current abilities (Saye and Brush, 2002; Wood et al., 1976). Scaffolds can be classified as hard scaffolds—pre-prepared instructional supports embedded in learning materials—or soft scaffolds, which are spontaneously provided by a teacher in response to learners’ needs (Saye and Brush, 2002). The following section will focus only on hard scaffolds.

One widely used approach is *worked examples*, which present learners with fully solved problems, allowing them to observe solution procedures without having to solve the task themselves (Renkl, 2014; Sweller et al., 2011). Worked examples consist of a problem statement, the solution steps, and the final solution itself (Kölbach et al., 2015). While effective in reducing cognitive load (Chen et al., 2023; Sweller et al., 1998, 2019), traditional worked examples may not fully foster deeper understanding if learners do not actively engage in self-explanations of the solution steps (Renkl, 2014; Roelle et al., 2023).

To address this limitation, integrating prompts into worked examples, encouraging learners to reflect on solution steps and generate self-explanations (Bannert, 2009; Roelle et al., 2023; Schmidt-Weigand et al., 2009). This represents an initial step towards more structured support that promotes active engagement over passive observation. However, even when worked examples include prompts, they often illustrate solutions for an example problem that differs from the task the learner is currently performing. Therefore, incremental scaffolds differ because they are designed to support learners directly in the context of the ongoing experiment.

Incremental scaffolds combine prompts and sample solutions in a staged manner (Hänze et al., 2010; Schmidt-Weigand et al., 2008). Originally, they are designed as folded paper-based material (A5 format), where the front page indicates the experimental step, the first unfolding reveals the prompt, and a further unfolding presents the corresponding sample solution. Prompts serve as aids for recalling knowledge or guiding task performance ranging from general questions to specific instructions (Bannert, 2009). They assume that learners already possess the relevant concepts or procedures but may not retrieve or apply them spontaneously (Bannert, 2009). Prompts present learners with cues or contextual information such as key concepts, principles, or relevant data that trigger cognitive engagement necessary for tackling a specific subtask (Schmidt-Weigand et al., 2008). They can consist of content-related as well as methodological guidance, for instance, by rephrasing the task, drawing attention to the starting point and the desired outcome, or activating prior knowledge (Hänze et al., 2010; Schmidt-Weigand et al., 2008). Students are first instructed to engage with the prompt, attempting to solve the tasks independently, before the corresponding sample solution is presented. The sample solution then provides verification feedback (Roelle et al., 2023), supporting comprehension, correct task completion, and potentially reducing uncertainty. Low-performing learners may rely on all levels of support, while high-performing learners might only consult the partial solutions to check their own approaches (Schmidt-Weigand et al., 2008).

Incremental scaffolds can be applied across different phases of the experimental process, such as formulating research questions, generating hypotheses, planning and conducting experiments, analyzing results, and drawing conclusions. By guiding learners gradually through each step, incremental scaffolds might reduce the cognitive load that can occur in open experimentation without support (Kirschner et al., 2006), while simultaneously promoting active processing and self-regulated learning (Hänze et al., 2010; Schmidt-Weigand et al., 2008, 2009).

In sum, incremental scaffolds can be seen as an evolution of worked examples (Arnold et al., 2017) because they retain the benefit of illustrating solution steps but add staged, task-specific prompts that directly support learners in the ongoing experiment, making the instructional support both structured and interactive. Further empirical findings and design variations are discussed in the subsequent section on the State of Research. To understand how incremental scaffolds influence learning, it is useful to consider the underlying cognitive and affective mechanisms. Extraneous cognitive load (eCL) reflects the effort spent on irrelevant or overly complex aspects of a task, while flow captures learners’ engagement when task demands match their skills. The following sections will therefore introduce the concepts of cognitive load and flow experience.

### 2.3 Cognitive load

Cognitive Load Theory (CLT) describes how the limitations of working memory constrain learning and emphasizes the importance of managing cognitive effort during complex tasks (Sweller et al., 1998, 2011, 2019). Cognitive load can be understood as the cognitive resources required in working memory to complete a specific task (Kalyuga, 2011; Sweller et al., 2011). If the cognitive demands of a task surpass the limited capacity of working memory, the resulting overload constrains information processing and thereby impairs both learning and performance (Jiang and Kalyuga, 2020; Kalyuga, 2011). Originally, a three-part structure of cognitive load was proposed, differentiating intrinsic, extraneous, and germane load. However, as already suggested by Kalyuga (2011), more recent studies confirm that intrinsic load largely overlaps conceptually with germane processes, leading to a two-factor model consisting of intrinsic and extraneous cognitive load only (Jiang and Kalyuga, 2020).

Intrinsic cognitive load reflects the inherent complexity of the material and varies with the degree of element interactivity and the learner’s prior knowledge (Ashman et al., 2020; Paas et al., 2010). Given that intrinsic load is intrinsically determined by the complexity of the content and the learner’s prior knowledge, it is largely unaffected by instructional design (van Merriënboer and Sweller, 2010). This is in contrast to extraneous load, which can be systematically influenced through the design and sequencing of learning materials (Sweller et al., 2019).

eCL arises from aspects of instruction that do not directly contribute to learning, such as poorly designed materials, redundant or distracting information, or unnecessary complexity of learning materials (Paas and van Merriënboer, 2020; Sweller et al., 2019). It can also occur during unguided problem-solving (Kirschner et al., 2006; Sweller et al., 1998). High eCL reduces the working memory resources available for processing essential information and therefore undermines learning outcomes (Paas and van Merriënboer, 2020). Thus, instructional designs play a key role in minimizing eCL. Scaffolding, including incremental scaffolds, provides learners with relevant cues and prompts, guiding them through the task while reducing extraneous cognitive demands and enhancing learning efficiency (Belland et al., 2013; Kirschner et al., 2006; Schmidt-Weigand et al., 2008). Given that intrinsic and extraneous load are additive (Sweller et al., 2011), the reduction of extraneous load is particularly relevant when learners are confronted with highly complex tasks or possess only limited prior knowledge (Paas and van Merriënboer, 2020). This highlights the necessity of designing instructional materials in line with CLT. Minimizing extraneous load not only improves learning efficiency but also frees cognitive resources, creating the conditions under which learners can experience flow, a state characterized by deep engagement and optimal challenge (Csikszentmihalyi, 1990, 2010).

### 2.4 Flow

Flow describes a psychological state in which learners are completely absorbed in an activity, experiencing a continuous, moment-to-moment engagement where their cognitive resources are fully concentrated on the task, allowing them to operate at their maximum capacity (Csikszentmihalyi, 1990, 2010; Csikszentmihalyi et al., 2014). Learners often lose awareness of time, fatigue, and other external factors while immersed in the task (Csikszentmihalyi, 1990, 2010). The emergence of flow depends on three interrelated conditions. First, there must be a balance between perceived skills and task demands, offering tasks that push learners to extend their current abilities while remaining within their skill limits. Second, learners benefit from clear proximal goals that help immediately focus their actions. Third, timely feedback is necessary to enable continuous adjustment and maintain engagement throughout the activity (Csikszentmihalyi et al., 2014; Nakamura and Csikszentmihalyi, 2014).

Csikszentmihalyi (1975) described the conditions for flow in what is known as the channel model of flow experience. The channel model emphasizes that flow only occurs within a limited zone where task demands and perceived skills are optimally aligned. If skills exceed demands, learners may experience underload, resulting in distraction or boredom. Conversely, if demands surpass abilities, overload can occur, triggering conscious control mechanisms and potentially anxiety, which hinders immersion (Ufer, 2020).

Since flow is closely linked to motivation and engagement (Csikszentmihalyi, 1990, 2010), tasks providing this optimal level of challenge may foster intrinsic motivation, sustained attention, and deeper cognitive processing. Incremental scaffolds can help create these conditions by guiding learners through complex tasks with staged prompts and partial solutions. By reducing eCL (Arnold et al., 2017; Kirschner et al., 2006) while preserving learner autonomy (Hänze et al., 2010), learning supports such as incremental scaffolds help maintain the balance between skill and challenge, thereby potentially facilitating the emergence of flow and enhancing the quality of the learning experience.

### 2.5 State of research

Empirical studies show that complex tasks such as open experimentation can cognitively overwhelm students, especially novice learners, when no adequate support is provided (Belland et al., 2013; Kirschner et al., 2006). Previous studies have shown that instructional support can help students handle complex tasks such as experimentation (Arnold et al., 2017; Großmann and Wilde, 2019; Stiller and Wilde, 2021; Kleinert et al., 2022; Hülsmann and Wilde, under review; Fleischer et al., 2023; Kirschner et al., 2006; Mustafa et al., 2021; Schmidt-Borcherding et al., 2013; Schmidt-Weigand et al., 2008, 2009; Wirth et al., 2008).

However, the effects of instructional support depend strongly on the type and amount of guidance provided. Worked examples provide fully guided solution steps and have been shown to reduce cognitive load (Chen et al., 2023; Sweller et al., 1998). Yet, overly directive guidance can limit learners’ active engagement (Chamberlain et al., 2014). In contrast, prompting provides cues or contextual information that activate students’ prior knowledge and stimulate cognitive engagement (Schmidt-Weigand et al., 2008). Research has shown that prompting can support students’ learning processes during experimentation (Wirth et al., 2008), and foster self-regulated learning (Bannert, 2009; Wirth, 2009) by encouraging learners to plan, monitor, and reflect on their own problem-solving processes.

Building on this idea, incremental scaffolds could be seen as an attempt to combine the advantages of worked examples and prompts. By integrating prompts and sample solutions within incremental scaffolds, learners are encouraged to generate self-explanations (Bannert, 2009; Roelle et al., 2023; Schmidt-Weigand et al., 2009) and receive feedback on their responses (Hänze et al., 2010; Schmidt-Weigand et al., 2008), which may lead to deeper processing and understanding (Renkl, 2014; Roelle et al., 2023).

Empirical findings indicate that incremental scaffolds can foster scientific reasoning (Arnold et al., 2017), knowledge acquisition (Großmann and Wilde, 2019; Stiller and Wilde, 2021; Hülsmann and Wilde, under review; Mustafa et al., 2021), motivation (Kleinert et al., 2022; Schmidt-Borcherding et al., 2013), and may also reduce extraneous cognitive load (Arnold et al., 2017). Despite these positive effects, most studies have examined incremental scaffolds as a whole. Consequently, it remains unclear which element—prompt or sample solution—causes the observed benefits. Moreover, findings regarding eCL are mixed. While Arnold et al. (2017) found a reduction in eCL through incremental scaffolds, other studies reported no such effect compared to unguided conditions (Hülsmann et al., 2024b, Hülsmann and Wilde, under review). In addition, other studies indicate that experimentation with recipe-like step-by-step instructions can lead to lower eCL than experimentation with incremental scaffolds (Schmidt et al., 2019). This suggests that the relationship between the degree of guidance and cognitive load is not yet conclusively understood, and that existing evidence on eCL remains inconclusive. These divergent findings highlight the need for further investigation of eCL. While previous research has examined the effects of incremental scaffolds on learners’ motivation (Kleinert et al., 2022; Schmidt-Borcherding et al., 2013), the potential for incremental scaffolds to support learners’ flow experience (Csikszentmihalyi, 1990, 2010) has not yet been investigated. Studying flow alongside eCL could therefore provide complementary insights into how incremental scaffolds influence not only cognitive aspects but also learners’ engagement and intrinsic involvement during complex experimentation tasks.

Given the complexity of experimentation and the goal of investigating the mechanisms of incremental scaffolds, this study deliberately focuses on extraneous cognitive load and flow experience. These constructs are theoretically well aligned with the assumed effects of scaffolding: extraneous cognitive load captures the efficiency of instructional guidance, while flow reflects the quality of learners’ engagement when task demands and skills are well balanced. Other potentially relevant variables, such as knowledge acquisition, were not included, as the focus here is on process-related mechanisms rather than outcome measures.

## 3 Research question and hypotheses

Experimentation in biology lessons is a demanding learning activity, as students must coordinate procedural, conceptual, and strategic aspects simultaneously, which can easily result in cognitive overload (Kirschner et al., 2006). Instructional guidance is therefore essential to make experimentation both cognitively manageable and engaging (Belland et al., 2013; Hänze et al., 2010; Kirschner et al., 2006; Schmidt-Weigand et al., 2008). Incremental scaffolds represent one promising form of such guidance: they provide stepwise support by first offering a prompt that stimulates students’ cognitive activation and reasoning, followed by a sample solution serving as verification feedback (Roelle et al., 2023) that ensures correctness and may reduce uncertainty (Hänze et al., 2007, 2010; Schmidt-Weigand et al., 2008). In contrast, students who receive only sample solutions lack this intermediate cognitive activation phase and may potentially approach experimentation in a more passive manner. Thus, the critical question is whether the prompts embedded within incremental scaffolds are the decisive element that fosters more efficient and engaging learning processes.

To evaluate this assumption, two constructs are particularly relevant. eCL indicates the extent of unnecessary cognitive effort caused by ineffective task designs and can be reduced through well-designed guidance (Paas and van Merriënboer, 2020; Sweller et al., 2019). Flow, in contrast, reflects the quality of the learning experience, capturing learners’ deep involvement when challenge and skills are well balanced (Csikszentmihalyi, 1990, 2010). Together, these constructs provide complementary perspectives on learning quality: while eCL represents the cognitive cost of task processing, flow captures the subjective experience of engagement. When eCL load is low, flow is more likely to occur, allowing learners to become fully absorbed in the task. Incremental scaffolds may further facilitate the emergence of flow, as they can contribute to establishing the core conditions identified as prerequisites for this state, namely, a balance between perceived skills and task demands, the presence of clear proximal goals, and the availability of immediate feedback (Csikszentmihalyi et al., 2014; Nakamura and Csikszentmihalyi, 2014). In contrast, the use of sample solutions mainly provides feedback, but may not explicitly support the balance between skills and task demands or the clarification of proximal goals.

Considering both constructs allows for a comprehensive investigation of how different forms of instructional support influence students’ learning processes. Against this background, the present study addresses the following research question: To what extent do incremental scaffolds, compared to sample solutions, influence students’ extraneous cognitive load and flow experience during experimentation in biology classes?

Based on the theoretical assumptions outlined above, it can be hypothesized:

Students who experiment with incremental scaffolds report:

(H1) a lower extraneous cognitive load than students who only experiment with sample solutions.

(H2) a stronger flow experience than students who only experiment with sample solutions.

## 4 Methods

### 4.1 Sample

The present study included 145 students (50.7% female, 49.3% male) from the seventh and eighth grades (*M*_Age_ = 12.87 years, *SD*_Age_ = 0.64 years) at two university-track secondary schools (*Gymnasien*) in North Rhine-Westphalia, Germany. The sample comprised six classes. Participants were randomly assigned at the class level to either the experimental group, which worked with incremental scaffolds during experimentation (*n* = 67), or to the control group, which only received sample solutions during the experiments (*n* = 78).

### 4.2 Study design

**Figure 1:**
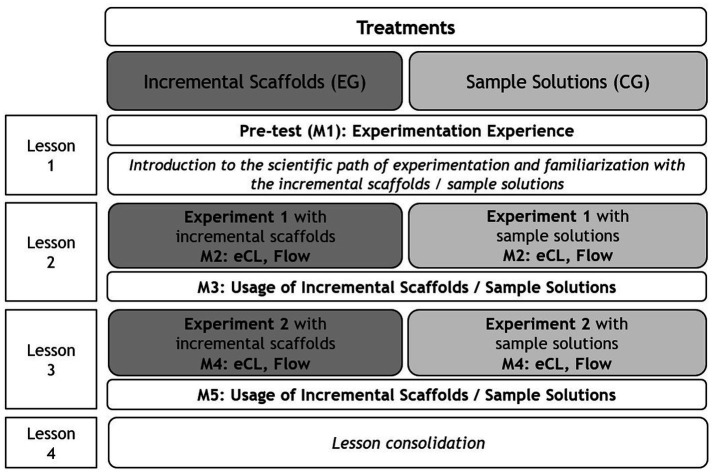
Overview of the study design, instructional conditions, and measurement points across all four biology lessons; shown are the experimental and control groups with their respective interventions and measurement points; EG, experimental group; CG, control group; eCL, extraneous cognitive load; M1–M5, measurement points.

The present quasi-experimental study comprised two conditions: an experimental group that used incremental scaffolds and a control group that received sample solutions only. Figure 1 illustrates the study design, including all measurement points (M1–M5). The teaching unit, which was implemented in regular biology classes and conducted by an advanced student teacher, consisted of four 45-min lessons, organized into two double periods (2 × 90 min).

In the first lesson, students were introduced to the scientific method of experimentation and familiarized with either incremental scaffolds or sample solution. In the following lessons, they conducted two model experiments on animal thermoregulation, with the final lesson devoted to discussing the results and consolidating key concepts. The experiments were identical across both groups; the only difference was the type of support provided (incremental scaffolds vs. sample solutions). Both types of support were provided in a paper-based format.

At the very beginning of the first lesson, students were asked about their previous experimentation experience. This procedure was intended to ensure group comparability, given that experimentation was part of the lesson. During the experimentation phases, short questionnaires embedded within the research protocols were administered to assess students’ flow experience and eCL (see section *Test Instruments* for details). Although both groups were required to use the provided support formats, students were asked to indicate whether they had used them at the end of both experimentation lessons (lesson 2 and 3).

### 4.3 Teaching and treatments

The first double lesson began with an introduction to the scientific method of experimentation. Using an example thought experiment on isopod habitat preferences, the experimental group practiced the use of incremental scaffolds, while the control group familiarized themselves with sample solutions. In the subsequent two lessons, students conducted two model experiments on animal thermoregulation, focusing on penguins and bats. Experimental materials were supplied in pre-arranged experiment boxes, ensuring that all necessary equipment was available to each group. Prior to the start of the experimental work, research questions and hypotheses were generated in a class discussion. Students then worked in groups of three to four to carry out the experiments. The experiments were deliberately designed as structured inquiry tasks. Given the research questions and hypotheses discussed in class as well as the materials, there was a single domain-appropriate experimental procedure and data collection required to validly address the research question.

Each experiment was structured using a paper-based research protocol, which guided students through three sub-steps: 1. Planning and Conduction, 2. Observation and Evaluation, and 3. Drawing a Conclusion. Students also recorded each of these steps directly on their research protocols. The research protocol began with a fictional research report, providing contextual information on the animal and its habitat. For example, the fictional research report on the bat experiment reads as follows:

“*It is often observed that bats gather in large, dense groups when temperatures are low. These large groups are referred to as colonies and can reach sizes of up to one thousand individuals. Within these colonies, bats often hang closely together in a roof-tile-like arrangement. This phenomenon is of particular interest and therefore raises the question of why bats assemble in such large groups under cold conditions. Since we have not yet investigated this ourselves, research findings will now guide us toward an explanation…*”

The research protocol already included specific task formulations for each step, for example in the planning and conduction phase: “What should be investigated and how? Plan an experiment using the provided materials, and make a sketch of the experimental setup.” In both groups, printed arrows on the side of the protocol indicated the availability of support for each of the three sub-steps (1. Planning and Conduction, 2. Observation and Evaluation, and 3. Drawing a Conclusion). In the experimental group, these arrows referred to incremental scaffolds, whereas in the control group they referred to sample solutions. The incremental scaffolds were based on the principles outlined by Hülsmann et al. (2024a) and adapted for this study with three incremental scaffolds rather than the proposed six to seven. This approach is in line with the implementation by Hülsmann and Wilde (under review). Students in the control group were provided with sample solutions only, which also followed the same three steps as the incremental scaffolds used in the experimental group. Accordingly, the sample solutions did not represent one possible solution among several equally valid alternatives, but illustrated the required experimental procedure and analysis for the given task. While students could differ in how they documented observations or measurements (e.g., using bullet points instead of a table), the underlying experimental logic and interpretation corresponded to the prompts and sample solutions. Students were therefore required to relate and abstract their own approach with respect to the structured procedure provided by the scaffolds.

**Figure 2:**
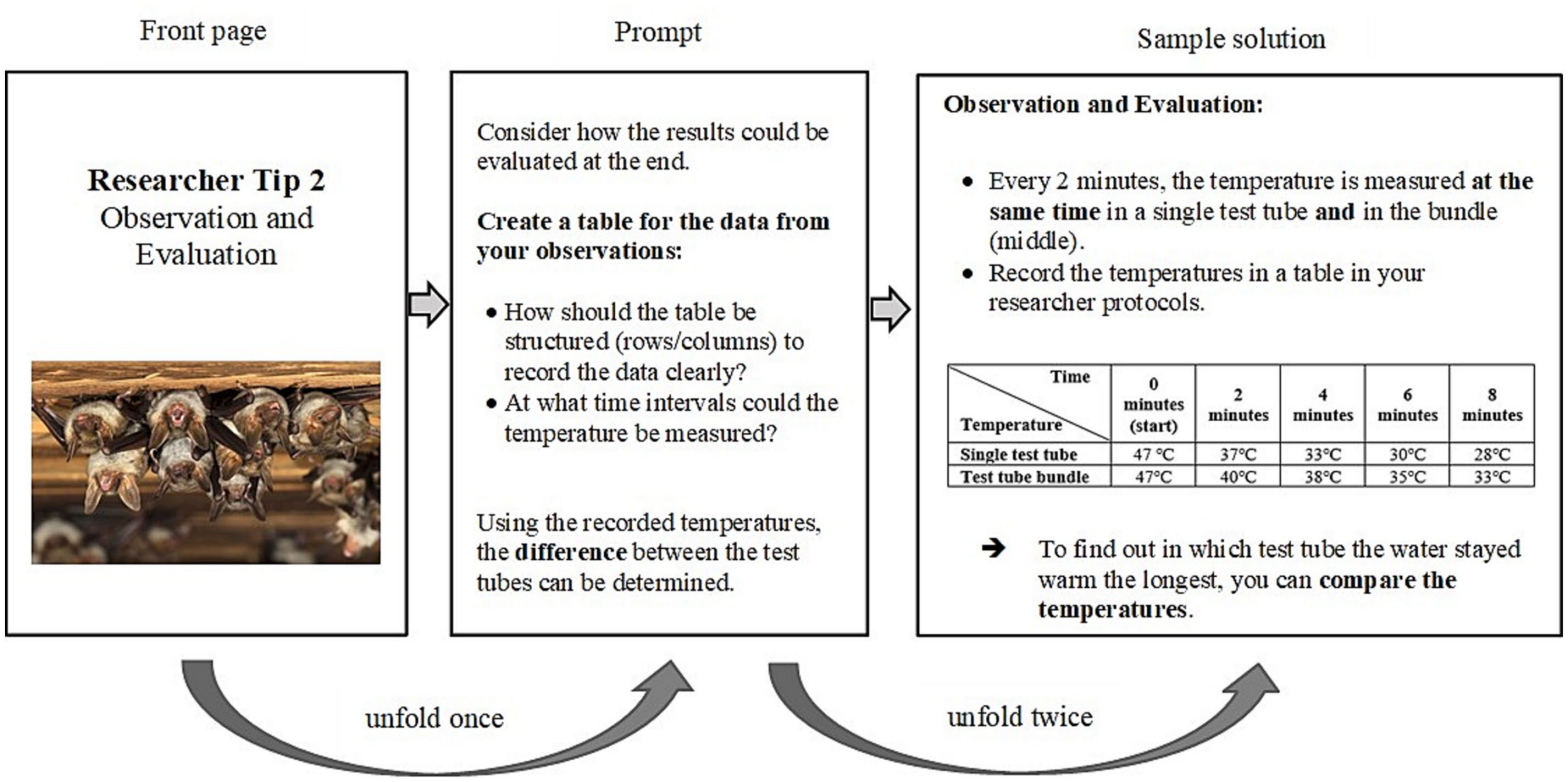
Example of an incremental scaffold in the observation and evaluation phase during the bat experiment. Shown is an incremental scaffold with a front page, a prompt and a sample solution, which are revealed stepwise. The complete version was used in the experimental group, whereas the control group only received the sample solution (without prompt).

Figure 2 provides an example of an incremental scaffold (always consisting of a prompt and a sample solution) used in the observation phase of the bat experiment. The control group, in turn, only received the depicted sample solution without the intermediate step of prompts. The final lesson was devoted to consolidation, reviewing the findings from the experiments and reinforcing students’ understanding of the key concepts related to animal thermoregulation.

### 4.4 Test instruments

At the beginning of the first lesson, prior to the intervention, students’ experimentation experience was assessed using the “Experimentation” scale for science education from the 2006 PISA studies (Frey et al., 2009), which comprises four items (e.g., “I have drawn conclusions from an experiment that I conducted myself.”; *α* = 0.81). Responses were recorded on a five-point scale ranging from 0 (‘never’) to 4 (‘in [almost] every lesson’).

During the experiments, eCL and flow experience were measured in-action, directly integrated into the research protocol immediately after the experimental phase, before students began data evaluation. eCL was assessed with the three-item subscale by Klepsch et al. (2017); e.g., “During the task, it was difficult to recognize and link the crucial information.”, using a five-point scale from 0 (“not true at all”) to 4 (“completely true”), showing acceptable internal consistency (M2: *α* = 0.64; M4: *α* = 0.75). Flow was measured using the 10-item *Flow Short Scale* by Rheinberg et al. (2003), which demonstrated good reliability (M2: *α* = 0.82; M4: *α* = 0.84).

After each experiment, students’ use of incremental scaffolds and sample solutions was recorded. This served as an implementation control to assess whether and to what extent participants used the assigned learning supports. Students indicated whether they had used the incremental scaffolds or sample solutions using a simple yes/no question. Although self-reporting may be influenced by factors such as recall errors or social desirability (Moosbrugger and Kelava, 2012), this approach offered a practical way to approximate actual usage under realistic classroom conditions.

### 4.5 Statistical analysis

To examine potential differences in students’ prior experimentation experience before the intervention, a univariate analysis of variance (ANOVA) was conducted. In addition, separate univariate ANOVAs were carried out for eCL at measurement points M2 and M4. Likewise, flow experience was analyzed using two separate ANOVAs at the same measurement points (M2 and M4).

## 5 Results

Table 1 presents the descriptive data on the use of incremental scaffolds and sample solutions across both experiments. In both the experimental and control group, a decrease in usage within the individual experiments can be observed.

**Table 1:** Descriptive overview of students reporting their use of incremental scaffolds in the experimental group and sample solutions in the control group in both experiments (yes-responses only).

	Experiment 1	Experiment 2
Incremental scaffold 1	76.2%	60.5%
Incremental scaffold 2	34.9%	20.4%
Incremental scaffold 3	34.9%	22%
Sample solution 1	70.3%	68.7%
Sample solution 2	32.9%	35.4%
Sample solution 3	32.4%	29.2%

Concerning the pre-test values of experimentation experience (EG: *M* = 2.00, *SD* = 1.01; CG: *M* = 1.91, *SD* = 1.03), there were no significant differences between the treatment groups [*F*(1, 136) = 0.256, *p* = 0.613], suggesting that students across groups started with comparable preconditions for experimentation.

Regarding eCL in the first experiment, the ANOVA only revealed small descriptive differences (EG: *M* = 1.13, *SD* = 0.70; CG: *M* = 1.22, *SD* = 0.66), but no statistically significant difference between groups [*F*(1, 130) = 0.572, *p* = 0.451]. In the second experiment, eCL significantly differed between the treatment groups [*F*(1, 117) = 4.548, *p* = 0.035, *η_p_^2^* = 0.037], indicating a small effect size (Cohen, 1988) with lower mean values for the experimental group (*M* = 0.83, *SD* = 0.57) compared to the control group (*M* = 1.10, *SD* = 0.74). Thus, the first hypothesis of a lower eCL in the experimental group can be partially confirmed, as it was supported in the second experiment but not in the first.

With regard to flow experience, students significantly differed between experimental (*M* = 2.39, *SD* = 0.66) and control group (*M* = 2.06, *SD* = 0.62) in the first experiment [*F*(1, 133) = 8.853, *p* = 0.003, *η_p_^2^* = 0.062], revealing a medium effect size (Cohen, 1988). In the second experiment, a significant difference between experimental (*M* = 2.62, *SD* = 0.60) and control group (*M* = 2.33, *SD* = 0.65) was also found [*F*(1, 117) = 6.436, *p* = 0.012, *η_p_^2^* = 0.052], indicating a small effect size (Cohen, 1988). The second hypothesis assuming a higher flow experience in the experimental group can therefore be confirmed.

## 6 Discussion

### 6.1 Cognitive load

The measurements for eCL only showed descriptive group differences in the first experiment, while the second experiment revealed significantly lower values for students who worked with incremental scaffolds. Hypothesis 1 can therefore only be partially confirmed. This pattern may suggest that the cognitive relief provided by incremental scaffolds unfolds only after learners have become familiar with their structure, as also indicated by previous research (Hülsmann and Wilde, under review; Hänze et al., 2010). While the present study did not directly examine this process, one plausible explanation is the following. Initially, students in the experimental group were likely cognitively occupied with understanding the interplay of prompts and solutions, whereas the control group using sample solutions may have faced less need for orientation because of the simpler format. This initial orientation may have required additional resources, which could have masked immediate cognitive relief effects. Moreover, experimentation as a learning activity is inherently complex (Kirschner et al., 2006), which may have kept students cognitively engaged in the task itself and might have initially overshadowed potential differences between support formats.

In the second experiment, however, students had possibly internalized the logic of the incremental scaffolds and could use the prompts more effectively. Effective usage may have reduced unnecessary search and orientation processes, directed attention to central aspects of the task, and broke down the experiments into smaller, more manageable steps (Hülsmann et al., 2024b). In this way, incremental scaffolds appeared to help learners focus on relevant information. In contrast, sample solutions, although seemingly simple, may have required students to integrate the presented results into their own reasoning without prior self-explanations or intermediate guidance (Roelle et al., 2023). While such solutions were immediately accessible and demanded no usage strategy, they did not provide adaptive or process-oriented support. This integration effort potentially consumed working memory resources without necessarily enhancing cognitive or affective learning processes. Incremental scaffolds thus might have offered cognitive relief by structuring the reasoning process rather than presenting complete solutions at once.

Moreover, this interpretation is supported by prior research on *worked examples*. Schmidt-Weigand et al. (2009) compared worked examples presented in a single, continuous format with an incremental presentation that unfolded step by step, yet without prompts, and a third condition that added preceding prompts to the incremental presentation. Their findings showed that an incremental presentation alone already reduced cognitive load compared to worked examples presented as a whole. Additionally, they found that when prompts are added to such incremental formats, learning benefits extend beyond cognitive relief and also include improvements in knowledge retention (Schmidt-Weigand et al., 2009). Their study did not examine incremental scaffolds identical to those in the present work, because they were designed for and applied to problem-solving tasks in physics solved in pairs rather than in group work during experimentation in biology classes. Nevertheless, Schmidt-Weigand et al.’s (2009) work points to the added value of combining an incremental presentation of worked examples with prompts that can reduce cognitive load and may actively guide learners through the reasoning process.

To conclude, the absence of significant group differences in the first experiment is likely due to an orientation phase in which students might had to familiarize themselves with the format of incremental scaffolds. Once this phase had passed, the prompts embedded within incremental scaffolds might have proved effective in reducing unnecessary cognitive load, underlining the importance of not only providing scaffolds but also allowing learners sufficient time to learn how to use them effectively (Hülsmann and Wilde, under review).

It should be noted, however, that other factors may also have contributed to the observed patterns of extraneous cognitive load. For example, the novelty effect of engaging in the experiments themselves, and the novelty effect of the support formats, or the selective use of specific scaffold steps by students could have influenced cognitive load measures. Both the novelty value of experimentation and the support formats may have decreased between experiments. Additionally, different usage patterns of the incremental scaffolds and sample solutions by the students may have had different effects on their perceived cognitive load. Finally, the results of the first experiment should be interpreted with caution, as the Cronbach’s alpha value of the eCL scale was only 0.64, which may limit the reliability of this measurement (Taber, 2018) and may be a reason why no differences in eCL between the treatments were found during the first experiment.

The observed decline in scaffold usage across steps and across both experiments may also help to contextualize the eCL findings. If later scaffold components were accessed less frequently, their potential to reduce extraneous cognitive load may not have been fully realized. This means that students may have experienced slightly higher eCL when accessing fewer scaffold components, because some procedural or interpretive support was missing. At the same time, reduced use of later supports may indicate that students experienced these phases as less cognitively demanding. This suggests that additional guidance was not perceived as necessary, which would support the claim of needs-oriented use (Hänze et al., 2010; Schmidt-Weigand et al., 2008). Consequently, the overall effect on eCL may reflect varying degrees of scaffold engagement. However, this variability in scaffolds engagement is not necessarily problematic, as the mean differences between the experimental and control groups already reflect these differing usage patterns.

### 6.2 Flow

Concerning Hypothesis 2, flow experience differed significantly between groups in both experiments, with students in the experimental group reporting higher values. This finding indicates that the benefits of incremental scaffolds with embedded prompts for engagement may manifest before measurable reductions in eCL. Flow theory (Csikszentmihalyi, 1975, 1990) posits that optimal experiences arise when task demands are well-matched to learners’ skills. The prompts within incremental scaffolds may support this balance: they activate prior knowledge, encourage independent reasoning, and promote reflective engagement (Kleinert et al., 2021), while the subsequent solution may reduce uncertainty. Even when students had not yet developed a full usage routine, the stepwise structure of the scaffolds may have conveyed a sense of orientation and controllability, thereby fostering a smoother learning process and stronger involvement. This could explain why motivational benefits (flow) became apparent from the first experiment, whereas measurable cognitive relief (eCL reduction) only emerged later.

From a theoretical perspective, this pattern may be related to the three preconditions of flow: a balance between skills and task demands, clearly defined proximal goals, and immediate feedback (Csikszentmihalyi et al., 2014; Nakamura and Csikszentmihalyi, 2014). Incremental scaffolds appear to address all three by structuring the experiment into manageable steps that maintain an optimal challenge by balancing skills and demands, directing learners toward proximal goals through prompts, and providing stepwise solutions serving as feedback that confirms students’ reasoning. In contrast, sample solutions mainly provide feedback on correctness but neither establish the skill–demand balance nor make goals explicit, which may limit their potential to foster flow.

Nevertheless, similar to considerations discussed for eCL, variability in scaffold use may also be relevant for interpreting the flow results. From a flow-theoretical perspective, both insufficient and excessive support could undermine optimal experience (Csikszentmihalyi, 1975, 1990; Csikszentmihalyi et al., 2014). Too little support may have led to overload, thereby reducing flow experience, whereas too much support may have lowered perceived and thus optimal challenge and resulted in boredom (Csikszentmihalyi, 1975, 1990). In contrast, selective and need-oriented engagement with different scaffolds may have enabled students to regulate task demands autonomously, maintain a balance between challenge and skill, and thereby sustain their flow experience. Rather than challenging the findings, this interpretation reinforces the notion that students’ adaptive scaffold use may have supported the conditions necessary for experiencing flow. Importantly, even if individual students varied in how they used the scaffolds, these variations are already reflected in the group means, which still revealed significant differences between experimental and control groups.

Interestingly, flow effects were observed even before reductions in extraneous load appeared, suggesting that flow may respond more immediately to the motivational and engagement-enhancing aspects of incremental scaffolds. While eCL reflects the efficiency of cognitive processing and may require some familiarization with the incremental scaffold format before reductions become apparent, flow captures learners’ subjective experience of involvement and challenge. Taken together, these findings indicate that incremental scaffolds may initially foster engagement and flow, and could contribute to more efficient cognitive processing, highlighting their complementary benefits for both cognitive and affective aspects of learning.

### 6.3 Limitations

While the findings provide valuable insights into the effects of incremental scaffolds, several limitations of the present study need to be considered. One limitation concerns the utilization of the provided supports. First of all, it should be noted that support use was assessed via a binary (yes/no) self-report item, which allows conclusions about whether supports were accessed, but not about the depth or quality of students’ engagement with them. However, the usage data revealed that in both the experimental and control groups, the first type of support (planning and conducting the experiment) was used most frequently, whereas the second (observation and evaluation) and third (drawing conclusions) were accessed less often (see *Results*, Table 1). This pattern was consistent across both experiments and closely mirrors results from a practice study on incremental scaffolds by Hülsmman and Wilde (under review), in which later support steps were also used less frequently. On the one hand, this reduced use of supports 2 and 3 could be seen as a limitation, since not all students engaged with the full range of provided guidance. On the other hand, the fact that the decline occurred in both groups makes the findings comparable and therefore less problematic for interpreting group differences.

Secondly, in line with the idea of scaffolding and fading (Puntambekar and Hubscher, 2010), the decreasing use of supports may not only reflect students’ selective engagement according to task demands but also reflect the intended fading process within scaffolding. As learners reach a higher level of competence, support is gradually faded out, so that guidance becomes less necessary and responsibility is increasingly transferred to the students themselves. Nevertheless, it highlights the importance of further emphasizing the role of data interpretation and conclusion-drawing in the scientific inquiry process (Hülsmann and Wilde, under review).

A third limitation is that the present design does not allow for isolating the specific contribution of prompts. Incremental scaffolds inherently combine prompts with corresponding solutions, whereas sample solutions provide only the latter. Since no condition was included in which students worked exclusively with prompts, it cannot be determined whether the observed benefits were primarily due to the prompts or the combination of prompts and solutions within the incremental scaffolds. However, the results suggest that the combination of both elements is more effective in terms of eCL and flow experience than solutions alone.

Fourth, all questionnaires used in this study were based on self-report measures. Although such instruments are susceptible to potential biases, including social desirability effects (Moosbrugger and Kelava, 2012) or memory inaccuracies, they offer a practical and feasible way to approximate students’ engagement under authentic classroom conditions. Alternative methods, such as eye-tracking (Kastaun et al., 2021) or structured classroom observations, could provide complementary information, but are often challenging to implement in typical school settings. In addition to these measurement-related limitations, statistical constraints also need to be considered.

Fifth, another limitation concerns the analytical treatment of the hierarchical data structure. Participants were assigned to conditions at the class level, resulting in students being nested within classes. Although multilevel modeling would, in principle, be an appropriate approach to account for such dependencies, the limited number of Level-2 units (i.e., classes) in the present study did not allow for reliable multilevel analyses (Hox et al., 2017). Therefore, univariate analyses of variance were applied. Future studies with larger samples and more clusters could consider multilevel approaches to account for nested data structures.

Lastly, the generalizability of the present findings is limited. The study was conducted with eighth-grade students from university-track secondary schools (*Gymnasien*) in biology classes, which restricts the extent to which the results can be applied to other age groups, school types, or subject areas. Moreover, the short-term nature of the intervention limits conclusions about long-term effects. While the study covered two consecutive experiments, it remains unclear whether the observed benefits of incremental scaffolds persist over time and translate into durable changes in learning and motivation.

Despite these limitations, the present study provides valuable insights into how the combination of prompts and sample solutions within incremental scaffolds can foster both cognitive efficiency and positive learning experiences. Building on these findings, the next section discusses practical implications for instructional design and outlines directions for future research.

## 7 Outlook

### 7.1 Educational implications

From a practical perspective, the results suggest that an incremental presentation of support in the form of prompts and solutions (incremental scaffolds) is a beneficial approach to foster both engagement and cognitive relief. Nevertheless, it may be advisable, especially for novice learners (Kirschner et al., 2006), to begin with more guided formats such as worked examples (e.g., Schmidt-Borcherding et al., 2013; Schmidt-Weigand et al., 2009) or recipe-like instructional support (Stiller and Wilde, 2023), before gradually moving toward incremental scaffolds. In this way, students can build familiarity with structured support before being required to regulate their own use of prompts and solutions. Furthermore, Hülsmman and Wilde (under review) propose using soft scaffolding (Saye and Brush, 2002) in form of teacher support in parallel with hard scaffolding in form of incremental scaffolds at the beginning, before only using incremental scaffolds. This intermediate format between soft (teacher support) and hard scaffolding (incremental scaffolds) may be particularly suitable for familiarizing students with the new adaptive learning support, since its benefits may require self-regulated learning skills itself (Hülsmman and Wilde, under review).

In this context, it should also be mentioned that at least in the initial phase, the use of incremental scaffolds should not be left entirely optional. Prior research indicates that when the choice of support use is fully delegated to learners, many struggle to adequately monitor their own learning processes and consequently fail to make optimal use of the available guidance (Ryan and Shin, 2011). At the same time, however, in line with the scaffolding principle of fading (Puntambekar and Hubscher, 2010), guidance should be gradually withdrawn and responsibility progressively shifted to the learners. This aligns with the adaptive nature of incremental scaffolds (Franke-Braun et al., 2008) and with the pedagogical goal of enabling learners to decide autonomously whether and when to make use of support (Hänze et al., 2010).

In addition, the findings on incremental scaffold use highlight the importance of emphasizing the entire experimentation process as a whole. While planning and conducting experiments are highly salient for students, equal weight should be placed on the careful evaluation of results and on drawing valid conclusions in relation to the research question and underlying hypotheses (Hülsmman and Wilde, under review). Strengthening this awareness may help learners to perceive experimentation not only as procedural execution but as a holistic process of procedural understanding.

## 7.2 Future research

Concerning empirical research, future studies should systematically examine how the number and type of prompts can be systematically altered. Additionally, it would also be worthwhile to explore digital formats of incremental scaffolds (e.g., Kleinert et al., under review; Fleischer et al., 2023) to test whether adaptive digital environments can further enhance their effectiveness. Also, in terms of diverse learner groups, these considerations could be meaningful since a digital presentation allows for more manifold features such as read-aloud functions, glossaries, videos or hyperlinks to additional sources (Kleinert et al., under review).

Another approach would be to separate the two scaffold components—prompts and solutions—by adding a treatment condition in which students receive prompts only. Such a design could clarify to what extent the observed benefits stem from the combination of prompts and solutions or whether prompts alone already provide substantial support. Still, it should be noted that the absence of feedback, inherent in a “prompts only” condition, might undermine positive self-explanation effects (Schmidt-Weigand et al., 2009).

Moreover, the present study focused primarily on flow experience and eCL as outcome variables. To broaden the perspective, future research should include additional affective factors such as intrinsic motivation, basic need satisfaction (autonomy, competence, relatedness) or interest, and examine how prompts within incremental scaffolds shape motivational and social learning processes. Prompts may also encourage more cooperative forms of interaction by stimulating dialogue about intermediate steps and strategies rather than merely focusing on final solutions. Such cooperation may strengthen students’ sense of relatedness, a key component of basic need satisfaction alongside autonomy and competence (Osterman, 2000; Ryan and Deci, 2017). In this way, prompts could not only facilitate cognitive engagement but also contribute to intrinsic motivation through fostering group connectedness and shared problem solving (Schunk et al., 2008). Comparing conditions with and without prompts may thus help to clarify whether these motivational and social benefits are specific to scaffold designs that integrate prompting elements.

Finally, exploring the applicability of incremental scaffolds to other subjects could be valuable, particularly for tasks that are sufficiently complex and follow a linear sequence of steps, as these conditions are crucial for the effectiveness of incremental scaffolds (Hänze et al., 2010). For example, incremental scaffolds might support students during experimentation in physics or chemistry, or in language classes for tasks such as text analyses that require a stepwise approach. Investigating these contexts would help to clarify to what extent incremental scaffolds can be transferred to other subject areas with differing disciplinary logics. More generally, to gain deeper insights into how students engage with incremental scaffolds, future studies could complement quantitative approaches with qualitative methods, such as structured classroom observations conducted by independent observers. This might allow for a more nuanced understanding of scaffolds use helping to examine the generalizability of the present findings.

## Data Availability

The datasets presented in this article are not readily available because this study is part of a qualification work for which further analyses are planned. Requests to access the datasets should be directed to marlina.huelsmann@uni-bielefeld.de.
